# PI3k and Stat3: Oncogenes that are Required for Gap Junctional, Intercellular Communication

**DOI:** 10.3390/cancers11020167

**Published:** 2019-02-01

**Authors:** Mulu Geletu, Zaid Taha, Patrick T. Gunning, Leda Raptis

**Affiliations:** 1 Department of Biomedical and Molecular Sciences, Queen’s University, Kingston, ON K7L 3N6, Canada; ztaha@ohri.ca (Z.T.); patrick.gunning@utoronto.ca (P.T.G.); raptisl@queensu.ca (L.R.); 2 Department of Pathology and Molecular Medicine, Queen’s University, Kingston, ON K7L 3N6, Canada

**Keywords:** gap junctions, phosphatidyl-inositol-3 kinase, Stat3, polyoma virus middle Tumor antigen, Src, electroporation in situ

## Abstract

Gap junctional, intercellular communication (GJIC) is interrupted in cells transformed by oncogenes such as activated Src. The Src effector, Ras, is required for this effect, so that Ras inhibition restores GJIC in Src-transformed cells. Interestingly, the inhibition of the Src effector phosphatidyl-inositol-3 kinase (PI3k) or Signal Transducer and Activator of Transcription-3 (Stat3) pathways does not restore GJIC. In the contrary, inhibition of PI3k or Stat3 in non-transformed rodent fibroblasts or epithelial cells or certain human lung carcinoma lines with extensive GJIC inhibits communication, while mutational activation of PI3k or Stat3 increases GJIC. Therefore, it appears that oncogenes such as activated Src have a dual role upon GJIC; acting as inhibitors of communication through the Ras pathway, and as activators through activation of PI3k or Stat3. In the presence of high Src activity the inhibitory functions prevail so that the net effect is gap junction closure. PI3k and Stat3 constitute potent survival signals, so that their inhibition in non-transformed cells triggers apoptosis which, in turn, has been independently demonstrated to suppress GJIC. The interruption of gap junctional communication would confine the apoptotic event to single cells and this might be essential for the maintenance of tissue integrity. We hypothesize that the GJIC activation by PI3k or Stat3 may be linked to their survival function.

## 1. Introduction

### Oncogenes and Gap Junctional Communication

Gap junctions are aqueous channels connecting the cytoplasm of adjacent cells that permit the passage of small molecules and ions [1,2]. Gap junctional, intercellular communication (GJIC) was long thought to play an important role in growth regulation. In fact, cancer was one of the first human pathologies to be associated with gap junction defects regarding both growth as well as metastatic potential [3], and a number of tumor lines and primary tumor cells were found to display a reduced GJIC [4,5,6]. Interestingly, the connexins, main gap junction proteins were later demonstrated to be able to regulate growth. This can occur through mechanisms that require cell to cell coupling, but also through GJIC independent mechanisms [7], since reintroduction of connexins in tumor cells can suppress growth without increasing coupling [8]. In the case of connexin43 (Cx43), the most studied connexin, growth suppression is likely caused by the C-terminal domain [9,10], and the effect can be through regulation of transcription, as well as cell to cell communication [11,12]. 

A number of oncogenes such as vSrc [13], vRas [14,15,16,17], mos [15] and others [18] were found to be able to interrupt GJIC. The mechanism of GJIC suppression by the prototype oncogene, Src and its effectors has been reported in several publications (reviewed in [13,19]). Briefly, at first the SH3 (Src homology-3) domain of Src binds a proline-rich area between P274 and P284 of Cx43, which brings the Src kinase domain in close proximity to Y265 of Cx43, which is then phosphorylated by Src. The phosphorylated Y265 offers a docking site for the Src, SH2 (Src homology-2) domain and this enhanced interaction causes the phosphorylation of Y247 of Cx43, which may contribute to GJIC reduction [20,21,22]. However, Src activates effector pathways such as PLCγ/PKC and Ras/Raf/Erk1/2, which are known to suppress GJIC in their own right [21,23,24,25,26]. Whether it is Src itself which blocks communication, or whether its effectors play a more important role is still controversial (reviewed in [27]).

Engagement of cadherins, cell to cell adhesion proteins, was shown to be required for gap junction formation [28]. In addition, results from our group and others showed that cadherin engagement activates two common effectors of a large variety of oncogenes including Src and Growth Factor Receptors, the phosphatidyl-inositol-3 kinase (PΙ3k [29]) and the Signal Transducer and Activator of Transcription-3 (Stat3 [30]). Since both PI3k and Stat3 are required for transformation and in an activated form can act as oncogenes, we and others set out to examine the effect of PI3k and Stat3 upon the gap junctional communication apparatus. The results revealed that, despite the general acceptance of oncogenes as GJIC suppressors, PI3k and Stat3, rather than suppress, they are actually required for the maintenance of communication and in an activated form they increase GJIC [4,31,32]. In this report we integrate some recent findings on the effect of PI3k and Stat3 upon GJIC.

## 2. PI3k as a Positive Regulator of Gap Junctional Communication

### 2.1. The Phosphatidylinositol-3 Kinase (PI3k)

The discovery of PI 3-kinases began with the identification of a phosphoinositide kinase activity present in immunoprecipitates of the polyoma virus middle Tumor antigen oncogene (mT). Later results demonstrated that this lipid kinase consists of two subunits: A catalytic, 110kDa (p110α or PIK3C), which phosphorylates phosphoinositides on the 3′ position of the inositol ring [33] and a regulatory, 85kDa subunit. PI3k is required for transformation of cultured cells by mT [34] and tumorigenicity in transgenic mice [35], while a large number of growth factor receptors and oncogenes bind to the p85 subunit and activate PI3k. Interestingly, engagement of cadherins also leads to PI3k activation [29].

Following expression of oncogenes such as mT or activated Src, or activation of membrane receptor tyrosine kinases (RTKs, e.g., EGFR and PDGFR), PI3k translocates to the membrane by binding through three SH2 (Src-homology-2) domains of p85, onto phosphorylated, pYXXM motifs of RTKs or adaptor proteins. Once on the membrane, PI3k initiates signalling cascades by generating the second messenger phosphatidylinositol (3,4,5)-trisphosphate [PI(3,4,5)P_3,_ known as PIP_3_] from phosphatidylinositol (3,4)-bisphosphate [PI(3,4)P_2,_ known as PIP_2_]. This provides docking sites for signalling molecules with plekstrin-homology (PH) domains such as Akt (reviewed in [36]). Akt is the most important PI3k effector and plays a pivotal role in cellular survival and metabolism, in addition to cell proliferation [37]. Akt’s pleckstrin homology domain binds directly to PtdIns(3,4,5)P3. Since this inositide is restricted to the plasma membrane, this binding drags Akt to the plasma membrane. Another kinase with a pleckstrin homology domain is the phosphoinositide-dependent kinase-1 (PDK1) which binds directly to PtdIns(3,4,5)P3. This binding triggers PDK1 translocation to the plasma membrane upon PI3k activation. Activated PDK1 then phosphorylates Akt on threonine 308, leading to partial activation of Akt. Full activation of Akt occurs upon phosphorylation of serine 473 by the TORC2 complex (mammalian target of rapamycin complex-2) of the mTOR protein kinase [38,39].

Evidence on the effect of PI3k upon GJIC is mainly from two systems: Section 2.2, PI3k interaction with and activation by mT and Section 2.3, direct PI3k activation through forced translocation to the membrane or mutation of the coding sequence.

### 2.2. mT, PI3k and GJIC

The study of mT has led to the discovery of important concepts in signal transduction. mT is a membrane-bound, 421-amino-acid protein which is able to transform cultured rodent fibroblasts and cause tumors following transgenic expression in mice [40]. mT transformation occurs through its association with members of the cellular Src protooncogene product family of tyrosine kinases, such as cSrc [41]. The mT:cSrc interaction stimulates cSrc enzymatic activity by forcing cSrc into an “open” configuration. Activated cSrc then phosphorylates several substrates including cSrc itself and mT at specific tyrosine residues. In this manner mT offers a scaffolding platform which attracts cellular signalling proteins [41,42]. As a result, the mT:pp60^c-Src^ complex offers the distinct advantage for signalling studies compared to activated Src in that each pathway can be individually inhibited, using mT constructs that are mutated at specific phospho-tyrosine residues which are responsible for activating specific pathways.

Three major tyrosine residues of mT provide docking sites for key signal transducers:

a. p-Y^250^ in a NPTpY^250^SVM motif recognizes the PTB domain of Shc, an adaptor which binds Grb2-sos and activates the Ras/Raf/Erk pathway [43,44].

b. p-Y^315^ in a pY^315^MPM motif recognizes an SH2 domain of p85, the adaptor subunit of PI3k [45,46,47,48,49,50,51].

c. p-Y^322^ recognizes the SH2 domain of Phospholipase-Cγ (PLCγ) [52].

**Figure 1:**
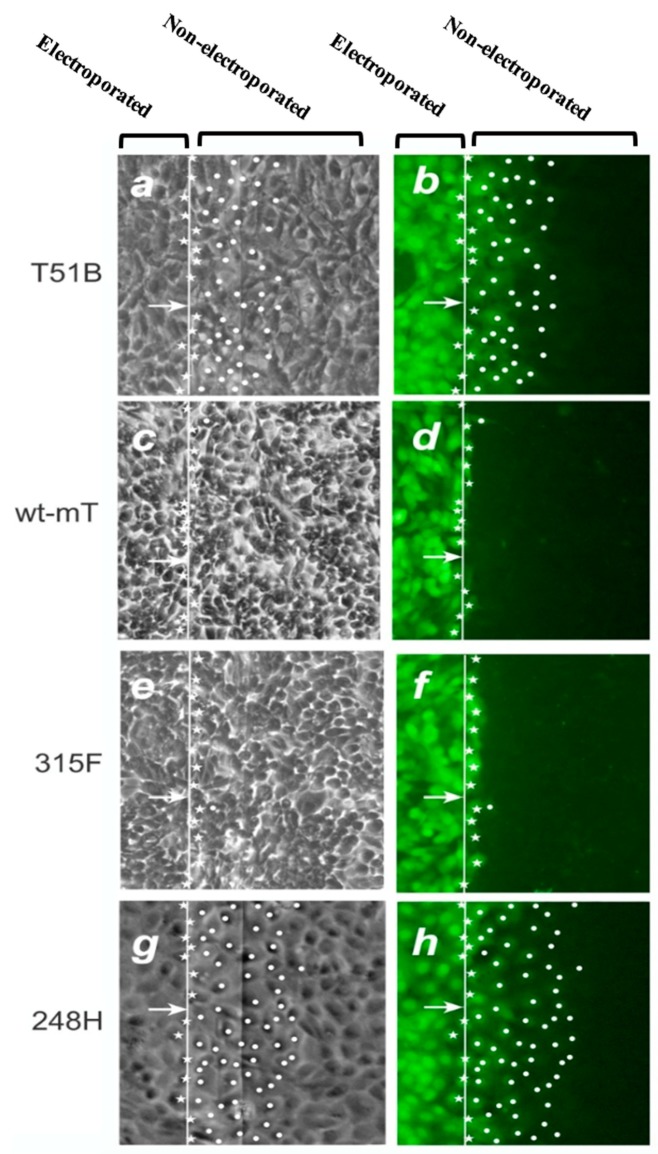
GJIC in T51B rat liver epithelial cells and derivatives expressing wt or mutant mT. GJIC was examined using a technique of in situ electroporation: The indicated cell lines were grown on a glass slide, part of which is coated with a thin layer (~800 Å) of electrically conductive and transparent Indium-Tin oxide, as shown at the top. The fluorescent dye, Lucifer yellow (LY) was added to the cells and introduced via an electric pulse which causes the formation of “pores” on the plasma membrane. The migration of LY to the neighboring, non-electroporated cells through the cells’ gap junctions is observed under fluorescence illumination (**b**,**d**,**f**,**h**) and offers a quantitative measure of GJIC (reviewed in [59]). (**a**,**c**,**e**,**g**): Phase-contrast images of the same fields. Stars indicate cells loaded with LY by electroporation. Dots denote cells where LY has penetrated through gap junctions. Arrows point to the edge of the electroporated area. Magnification: 240x. Note the extensive GJIC of T51B-248H cells. (From [31], reproduced with permission).

**Figure 2:**
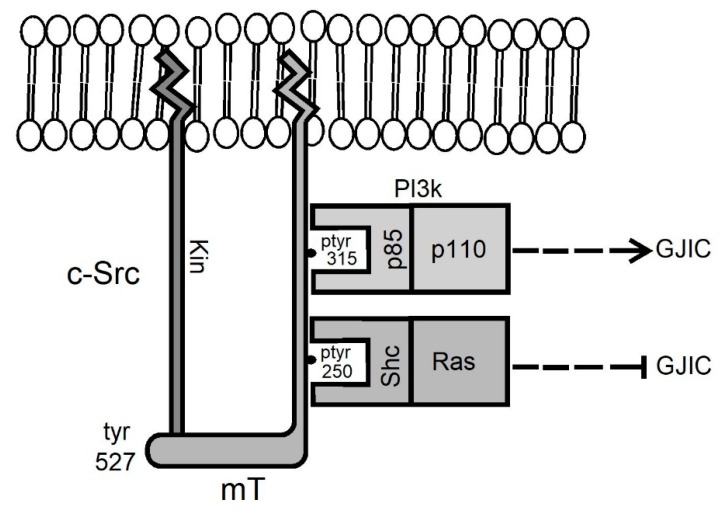
Model of the effect of the Ras/Erk vs. the PI3k/Akt pathways. mT binds to, activates and is phosphorylated by Src family kinases. The mT-pY^250^ site binds the Shc adaptor which triggers activation of the Ras/Erk pathway, leading to GJIC suppression. The mT-pY^315^ site binds the p85 regulatory subunit of PI3k, triggering activation of Akt, leading to GJIC increase. Upon wt-mT expression the Ras pathway prevails with elimination of communication as a result. Interestingly, neoplastic transformation by mT requires both pathways. Not shown: mT-pY^322^ activates PLCγ and PKC, which suppresses GJIC.

Early data demonstrated that even low levels of wild-type (wt) mT can effectively suppress GJIC in mouse fibroblasts [53,54]. To examine the effect of each of the two pathways emanating from mT separately, tyrosine to phenylalanine mutants deficient in binding p85 (315F) or Shc (250F), respectively, were stably expressed in the rat liver epithelial cell line T51B which normally has extensive GJIC [31]. The results revealed that the 250F mutation which abrogates Shc binding [43,55,56] abolishes the ability of mT to interrupt GJIC, indicating that Y^250^ phosphorylation (and presumably Ras/Raf/Erk activation) is in fact a key to GJIC suppression by mT [31]. In sharp contrast, the 315F mutant was just as effective as wt-mT in reducing gap junctional communication (Figure 1e,f), indicating that Y^315^ is not required for GJIC suppression. Therefore, activation of the Shc/Grb2/Ras pathway (through pY^250^ which is intact in 315F-mT) appears to be sufficient to drive the dramatic reduction in gap junctional communication [31]. The importance of the Y^250^, Shc binding site, rather than the Y^315^, in GJIC suppression is further supported by the fact that a mT mutant which activates PI3k/Akt more than wt-mT (mutant 248H where binding of Shc is impaired, but a secondary PI3k binding site is intact [57]) has actually higher GJIC than the parental T51B cells (Figure 1g,h vs. Figure 1a,b). Taken together, these findings reveal that PI3k activation by mT increases gap junctional communication. This increase can occur even in the face of activation of PLCγ/PKC, a known GJIC suppressor, and despite the fact that the 248H-mT mutant does activate the Src kinase as much as wt-mT [58], as long as the GJIC-suppressing, Ras/Raf/Erk pathway is not activated. In the presence of high Ras activity however, as upon wt-mT expression, the Ras GJIC-suppressive effect prevails, with gap junction closure as a result (Figure 2).

### 2.3. PI3k Mutations, Membrane Translocation and GJIC

PI3k inhibition with two different inhibitors (wortmanin or LY294002) resulted in a dramatic reduction of GJIC in T51B cells, pointing to a positive role of PI3k upon gap junctional communication in this system [31]. 

Examination of the effect of hyperactive transforming PI3k was performed through expression of two mutants of the catalytic subunit of PI3k, p110α (E545K and H1047R) found in a number of cancers [36], and a mutant with a myristyl group addition which renders it membrane-bound and constitutively active [60]. All three mutants that trigger a dramatic increase in Akt-pS473 levels [31] were found to increase gap junctional communication as well [31]. Taken together, the above results indicate that, despite its transforming effect, PI3k plays a positive role in the maintenance of gap junction function in this system.

In spite of extensive efforts, the effect of Akt upon GJIC of different cellular systems is unclear. Akt-mediated phosphorylation was shown to stabilise membrane-localised Cx43 [61,62,63], and to be required for the maintenance of steady-state Cx43 levels and GJIC in osteoblasts [64]. However, it was also shown that Akt1 (but not Akt2 or Akt3) actually participates in the disruption of gap junctions caused by activated Src in rodent fibroblasts [65]. In addition, PI3k reduced Cx43 levels and GJIC in a model of cerebral ischemia/reperfusion injury in rats [66], while PI3k was found to have both positive and negative effects upon GJIC in *Xenopus* oocytes [25]. In this system, PI3k-p110α co-expression increased Cx50-, but not Cx46-mediated gap junction coupling [67]. Since in T51B cells PI3k inhibition abolishes GJIC, while PI3k activation by 250F/248H-mT, membrane translocation or mutation increases GJIC, it appears that PI3k plays a positive role upon gap junctional communication in this system. It is possible that in these cells PI3k is activating all three Akt isoforms, so that the net effect is a GJIC increase. Alternatively, PI3k may promote nuclear accumulation of β-catenin which is known to stimulate Cx43 expression [68].

## 3. Stat3 as a Positive Regulator of Gap Junctional Communication

### 3.1. The Signal Transducer and Activator of Transcription-3 (Stat3)

Stat3, a member of the STAT family of transcription factors, is normally inactive in the cytoplasm of quiescent cells. Following stimulation of cytokine receptors especially of the IL6 family, certain RTKs, or oncoproteins such as Src, Stat3 is phosphorylated at a critical Y-705 by the associated Jak or Src kinases. Reciprocal SH2-pY interactions follow leading to dimerization, nuclear translocation and homing of the complex towards a specific sequence (TTCNNNGAA) on the promotors of target genes [69]. Stat3 activates the transcription of genes involved in cell division such as *myc.* However, Stat3 is also a potent cell survival signal that acts through a number of pathways: (1) Transcriptional upregulation of genes such as *bcl-xL*, *Mcl-1* and *survivin*; (2) transcriptional downregulation of the tumor suppressor p53 [69,70,71]; (3) transcriptional upregulation of the oxygen sensor HIF1α (hypoxia inducible factor-1α) transcription factor [72]; (4) In a transcription-independent manner, through an effect of Stat3 upon the mitochondria: Stat3 is also phosphorylated on S727 downstream of a number of stimuli that trigger MAP kinase activation, such as Ras signalling or stress [73,74]. Stat3-S727 localizes to the mitochondria where it enhances the activity of the electrotransfer chain complexes and increases glycolysis, thus promoting survival. Furthermore, Stat3-pS727 opposes the mitochondrial permeability transition pore, thereby inhibiting apoptosis even further [72,75,76]. 

Stat3 is found to be overactive in a number of cancers and to be required for transformation by a number of oncogenes such as Src [77,78,79]. Interestingly, substitution of two cysteine residues within the C-terminal loop of the SH2 domain of Stat3 (A661C and N663C), which renders Stat3 constitutively dimerized and active (Stat3C) is sufficient to induce neoplastic transformation of cultured mouse fibroblasts [80]. This observation reveals an etiological role for Stat3 in neoplasia.

Our lab and others recently demonstrated that, besides growth factors and oncogenes, confluence of a large variety of cultured cells induces a dramatic surge in Stat3, pY^705^ phosphorylation and activity ([81,82,83,84,85,86,87], reviewed in [88]). It was later shown that engagement of a number of cadherins, as occurs through confluence, triggers a surge in protein levels and activity of the small GTPases, Rac and Cdc42 [86,87,89,90,91]. Rac activation increases the secretion of IL6 family cytokines and autocrine activation of Stat3 ([86], reviewed in [30,88]). The importance of Stat3 in survival is demonstrated by the fact that Stat3 inhibition in Src-transformed, or non-transformed cells grown to high confluence induces apoptosis, not simply reversion of the cell to a normal phenotype [78,79,92]. The survival effect of Stat3 may be the reason for the resistance of tumor cells to chemotherapeutic drugs and targeted therapies when grown to high but not low densities in culture [93].

### 3.2. Stat3 Inhibition Eliminates GJIC While Stat3C Increases GJIC

Evidence on the effect of Stat3 upon GJIC stems mainly from Src-transformed, rodent cells as well as from human lung carcinoma lines and expression of a mutationally activated form of Stat3.

Ras pathway inhibition restores GJIC in vSrc-transformed, rat 3Y1 cells [13,94], while in non-transformed fibroblasts, Ras downregulation increased GJIC [14]. In sharp contrast to Ras inhibition however, pharmacological or genetic Stat3 inhibition in the rat liver epithelial line T51B transformed by activated Src (line T51B-Src), through the CPA7 inhibitor [95] or siRNA expressed with a retroviral vector did not restore GJIC [32]. That is, contrary to high Ras, high Stat3 activity, which could be, at least in part, due to high Src activity levels, cannot be responsible for the lack of junctional communication in T51B-Src cells.

Examination of the effect of Stat3 inhibition in the parental T51B line showed that treatment with CPA7 or siRNA expression essentially abolished GJIC [32]. Therefore, rather than increasing GJIC, as would be expected based on Stat3’s neoplastic properties, Stat3 inhibition eliminates junctional permeability, indicating that Stat3 activity is actually required for gap junction function in normal epithelial cells which display extensive GJIC. Results also demonstrated that, besides GJIC, Stat3 is also required for the maintenance of Cx43 protein levels as well [32,96].

**Figure 3:**
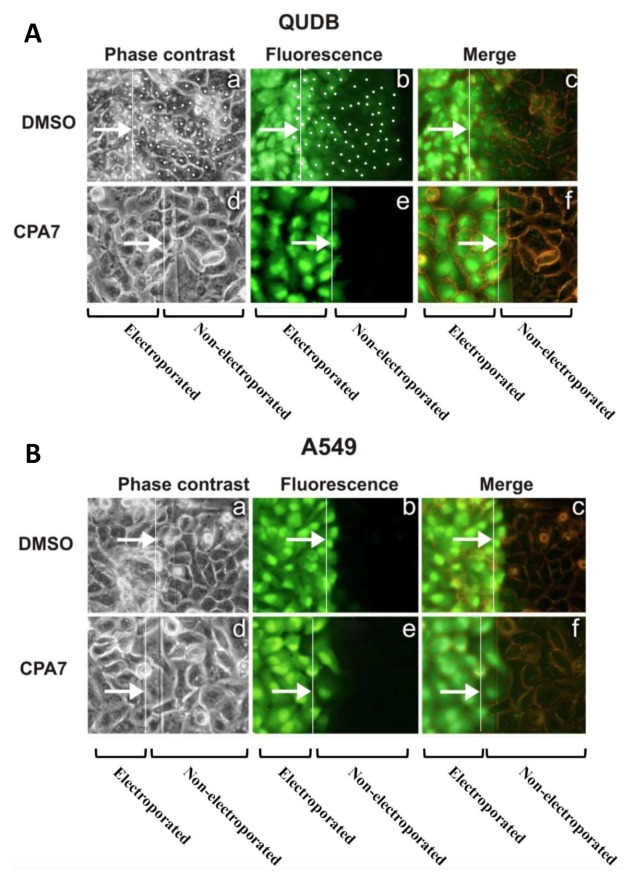

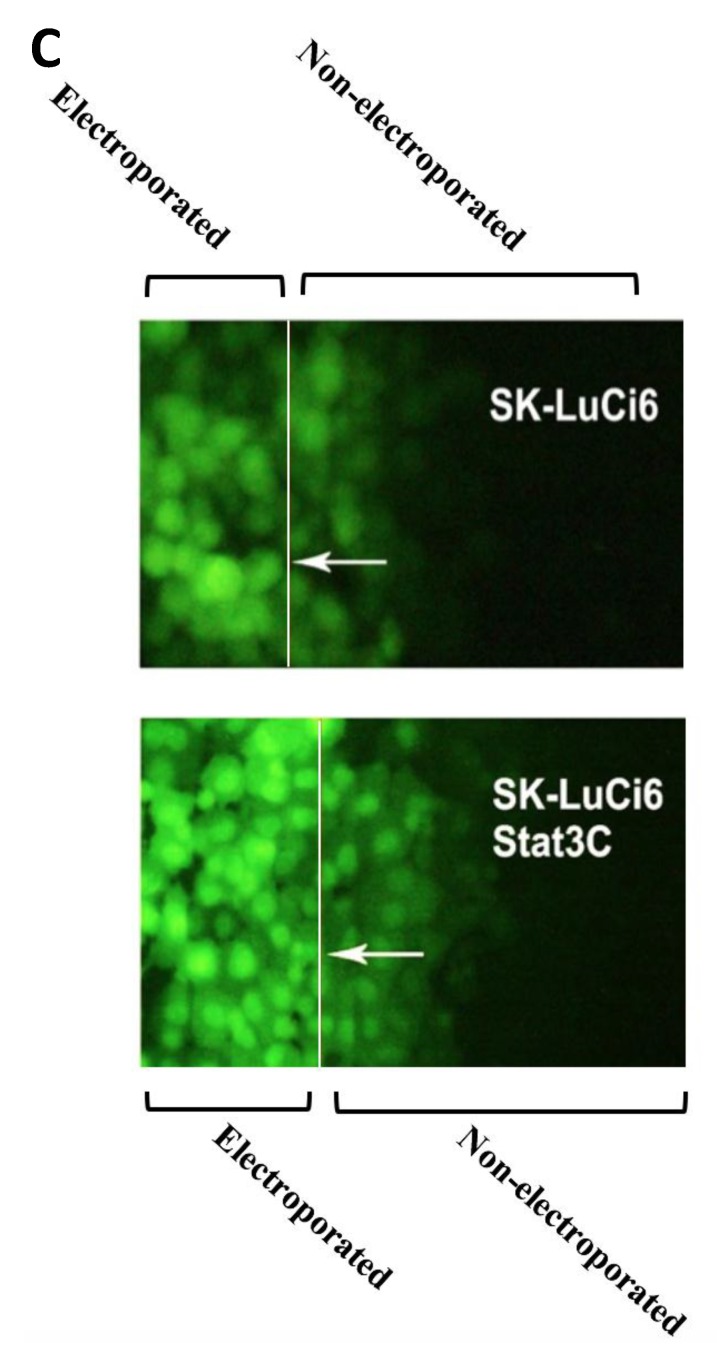
(**A**) Stat3 downregulation eliminates gap junctional permeability in human lung carcinoma QU-DB cells. LY was electroporated into QU-DB cells following treatment with the DMSO carrier alone (a–c), or the Stat3 inhibitor CPA7 (d–f). Cells from the same field were photographed under fluorescence (b,e) or phase contrast (a,d) illumination. Cells at the edge of the conductive area which were loaded with LY through electroporation were marked with a star, and cells at the non-electroporated area which received LY through gap junctions were marked with a dot [53]. Arrows point to the edge of the electroporated area. (c,f) Overlay of phase-contrast and fluorescence. Magnification: 240×. Note the extensive gap junctional communication in (b). (From ref. [4], reproduced with permission). (**B**) Stat3 downregulation does not increase gap junctional permeability in human lung carcinoma A549 cells. Same as above, A549 cells. Note the absence of GJIC, even after Stat3 downregulation (e). (From ref. [4], reproduced with permission). (**C**) Stat3C increases GJIC. LY was electroporated into SK-LuCi6, lung carcinoma cells before (top panel) or after (bottom panel) expression of Stat3C with a pBabe-puro-Stat3C retroviral vector. Note the increase in dye transfer following Stat3C expression. Arrows point to the edge of the electroporated area (Figure not previously published).

These results from rat epithelial cells were recapitulated in human non-small cell lung carcinoma lines (NSCLC). In fact, an inverse relationship between Src activity levels and GJIC was noted; in five lines with high Src activity (A549, SKLu-1, CaLu-1, SW900, CaLu-6), GJIC was absent (e.g., A549, Figure 3B, panel a–c), while two lines with low Src levels (QU-DB and SK-LuCi6) had extensive GJIC (e.g., QUDB, Figure 3A panel a–c), similar to non-transformed, immortalized lung epithelial cells [4]. Interestingly, Stat3 inhibition in any of the NSCLC lines expressing high endogenous Src activity levels, or in cells where Src was exogenously transduced, did not restore GJIC. On the contrary, Stat3 downregulation in immortalized, non-transformed lung epithelial cells or in the NSCLC lines displaying extensive GJIC actually suppressed junctional permeability, in a manner similar to the T51B/T51B-Src system (Figure 3A(d–f),B(d–f)), pointing to a positive role of Stat3 upon GJIC. Interestingly, expression of the constitutively active Stat3 mutant, Stat3C, in SK-LuCi6 cells increased GJIC (Figure 3C), despite the fact that Stat3C is capable of neoplastic transformation [80].

Further examination of additional lung cancer lines revealed a greater level of complexity [97], indicating that other, Src-independent factors must be responsible for GJIC suppression. Still, in all cases high Src levels, either endogenous or exogenously expressed, eliminated GJIC, while Stat3 inhibition eliminated junctional permeability in any of the lung cancer lines examined, in agreement with data from mouse or rat fibroblasts or epithelial cells. Taken together, these results show that, unlike Ras, Stat3 is actually required for, rather than suppressing junctional permeability.

## 4. Discussion

Neoplastic transformation is invariably accompanied by GJIC reduction [4,97]. In fact, several lines of evidence point to activated Ras as GJIC suppressor: Mutationally activated forms of Ras can suppress GJIC [14,15], and this reduction could be effected at lower levels than the levels required for full neoplastic conversion by this oncogene, pointing to an exquisite sensitivity of gap junction function to Ras signalling [17]. In addition, despite the fact that the prototype oncogene, Src, can suppress GJIC through direct phosphorylation of connexin-43 at Y247 and Y265 [98,99], or indirectly through activation of the Protein kinase C [23,100,101,102,103], GJIC suppression by vSrc or mT requires Ras signalling [16], and can also occur by a mechanism that is independent from Erk action, at least in 3Y1 rat fibroblasts [94]. However, despite the fact that the Ras, PI3k/Akt and Stat3 pathways are neoplastically transforming and are often co-ordinately regulated by Growth Factors or oncogenes, inhibition of PI3k or Stat3 activity does not restore junctional permeability in cells transformed by activated Src [4] or mT [31]. In the contrary, PI3k or Stat3 inhibition eliminated GJIC even in normal fibroblasts or epithelial cells [32], or certain lung cancer lines which have extensive GJIC [4]. These findings stress the role of PI3k and Stat3 as positive regulators of GJIC. Thus, it appears that oncogenes such as Src have a dual role upon GJIC; acting as inhibitors of communication through direct or indirect phosphorylation of Cx43, and as activators, through activation of PI3k and Stat3. In the presence of high Src activity the inhibitory functions prevail so that the net effect is gap junction closure.

Since neoplastic transformation leads to inhibition of junctional communication, the question of the transforming abilities of the two pathways, Ras and PI3k is next. In fact, both the Ras and PI3k pathways were shown to be required for full neoplastic transformation by mT [34]. Still, the fact that mutant 248H-mT which activates PI3k/Akt more than wt-mT [57] has actually higher GJIC than the parental T51B cells (Figure 1g,h vs. Figure 1a,b), while expression of activating mutations of p110α increases GJIC, reinforce the conclusion that PI3k is able to increase GJIC, despite its transforming potential. The fact that activation of the Ras pathway alone (mutant 315F-mT) is not sufficient for anchorage independence or tumorigenicity [104] indicates that the interruption of gap junctional communication by the Y^250^-Ras/Erk pathway can occur in the absence of full neoplastic conversion and the concomitant changes in cell shape in this system.

PI3k and Stat3, two evolutionarily very different oncogenes, both constitute potent survival signals. The question therefore arises as to whether the effect of PI3k or Stat3 upon GJIC is linked to their survival function.

Apoptotic death of adherent cells is associated with dramatic shape changes and a reduction in the area of cell to cell contact, which would lead to a disruption of gap junctions. Previous data demonstrated that apoptosis triggered by treatment with etoposide, cycloheximide or puromycin leads to a loss of GJIC, probably due to caspase-3-mediated degradation of Cx43 [105]. In fact, Akt is known to promote cellular survival through multiple mechanisms ([27,64,106,107], reviewed in [37]). Therefore, apoptosis inhibition due to PI3k/Akt activation would increase Cx43 levels and gap junction function. Similarly, Stat3 activates a number of anti-apoptotic genes, such as survivin [71], Bcl-xL and Mcl-1 [108]. Stat3 inhibition in cells grown to high densities was shown to trigger apoptosis in non-neoplastic mouse fibroblasts and epithelial cells as well as breast cancer lines [92], which is accompanied by a dramatic reduction in Cx43 levels and GJIC [4,32]. Experiments regarding GJIC levels following inhibition of specific, apoptosis-linked effectors of PI3k or Stat3 may definitively answer these questions.

## 5. Conclusions

Despite the fact that Ras, PI3k and Stat3 are generally growth promoting, required for transformation by a multitude of oncogenes, and in an activated form can act as oncogenes in their own right, PI3k and Stat3 are not transmitting signals leading to GJIC suppression. In the contrary, the available evidence reveals a dramatic difference in the response of GJIC to the three pathways emanating from many oncogenes and jointly required for full neoplastic conversion; PI3k and Stat3 actually promote GJIC. Therefore, it is tempting to speculate that neoplastic transformation requires Ras/Erk as well as PI3k/Akt, Stat3 and survival, while a survival function is actually required for the maintenance, rather than suppression, of GJIC. When all three pathways are activated then the Ras pathway prevails, with GJIC suppression as a net result. Upon PI3k or Stat3 inhibition, the interruption of GJIC would confine the apoptotic event to single cells and this might be essential for the maintenance of tissue integrity. This novel role of PI3k and Stat3 may be an important regulatory step in the progression of tumours that exploit such a pathway. In fact, a correlation was noted between nuclear Stat3 levels and Cx43 and Cx26 in grade 1 and 2 uterine endometrioid adenocarcinomas, while this association was progressively weaker as the tumors dedifferentiated to G3, presumably due to the activation of additional oncogenes [109].

**Figure 4:**
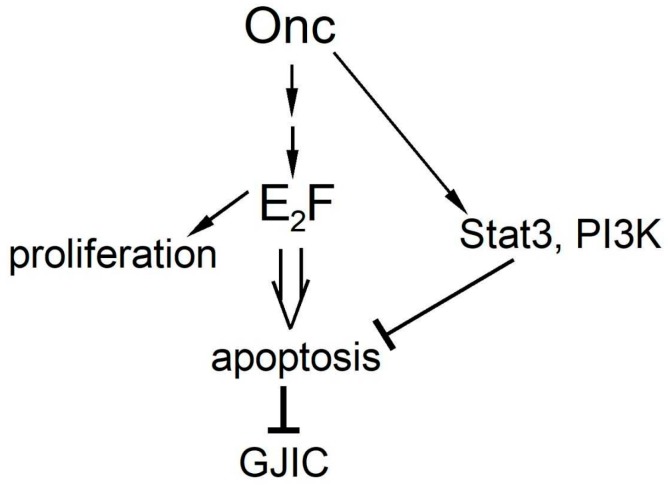
Model on PI3k and Stat3, apoptosis and GJIC. A number of oncogenes (Onc) activate the transcription factor E2F family, through a number of steps [111]. E2F, in turn, can induce apoptosis through p53-dependent and independent pathways. Interestingly, the oncogene also activates PI3k and Stat3 which block apoptosis, so that neoplastic transformation can occur. Upon PI3k and Stat3 inhibition however, apoptosis will suppress GJIC.

**Significance**: A large number of oncogenes activate the transcription factor E2F family, which targets genes involved in cell proliferation. Paradoxically however, E2F by default activates genes involved in apoptosis [110]. Certainly, the oncogene(s) or E2F itself also activate genes such as for PI3k and Stat3 which block apoptosis, so that transformation can occur. Still, inhibition of PI3k or Stat3 triggers apoptosis of tumor cells specifically due to their high E2F levels, rather than merely growth retardation (Figure 4). Although apoptosis of tumor cells is highly desirable in a clinical setting, the GJIC reduction occurring would prevent the diffusion of the inhibitory drugs to neighboring cells, a fact which should be taken into account in drug design.

## Abbreviations

Erk1/2	Extracellular signal regulated kinase 1 and 2
GJIC	Gap junctional, intercellular communication
ITO	indium Tin Oxide
LY	Lucifer yellow
mT	middle tumor antigen of polyoma virus
myr-p110	myristylated, p110 catalytic subunit of PI3k
PI3k	Phosphatidylinositol-3 kinase
p110	catalytic subunit of PI3k
PDK1	phosphoinositide-dependent kinase-1
PH	plekstrin-homology
PKC	Protein kinase C
PLCγ	Phospholipase-C gamma
PTB	phosphotyrosine binding domain
Ras	Rat sarcoma
Ser	serine (or S: serine)
SH2	Src-homology-2 domain
SH3	Src-homology-3 domain
Shc	Src-homology-2 domain-containing transforming protein
TORC2	Target of Rapamycin complex 2.
Tyr	tyrosine (or Y: tyrosine)
wt	Wild-type
